# Supplementary material for: Dealing with Headache: Sex Differences in the Burden of Migraine- and Tension-Type Headache
Source: Brain Sci. 2021 Oct 5;11(10):1323. doi: 10.3390/brainsci11101323 (PMC8534023; doi:10.3390/brainsci11101323)
Supplement: Supplementary file 1 [file brainsci-11-01323-s001.zip › brainsci-1388669-supplementary.pdf]

Table S1. Burden of headache of participants with TTH.

	all	females (%)	males (%)	Chi square	P value
	161	96 (59.6)	65 (40.4)		
**Problems in love life due to headache in the past 3 months**					
**No**	116 (82.86)	65 (78.31)	51 (89.47)	2.963	0.085
**Yes**	24 (17.14)	18 (21.69)	6 (10.53)		
	21 n.r.	13 n.r.	8 n.r.		
**Do you avoid telling people that you have headaches?**					
**No**	103 (72.54)	57 (67.86)	46 (79.31)	2.259	0.133
**Yes**	39 (27.46)	27 (32.14)	12 (20.69)		
	19 n.r.	12 n.r.	7 n.r.		
**Do you feel that your family and friends understand and accept your headaches?**					
**No**	11 (7.80)	7 (8.43)	4 (6.90)	0.112	0.738
**Yes**	130 (92.20)	76 (91.57)	54 (93.10)		
	20 n.r.	13 n.r.	7 n.r.		
**On that day, was there anything you could not do or did not do because you wanted to avoid getting a headache?**					
**No**	117 (86.03)	67 (83.75)	50 (89.29)	0.840	0.359
**Yes**	19 (13.97)	13 (16.25)	6 (10.71)		
	25 n.r.	16 n.r.	9 n.r.		
**On that day, were you anxious or worried about your next headache episode?**					
**No**	131 (94.93)	75 (92.59)	56 (98.25)	2.220	0.136
**Yes**	7 (5.07)	6 (7.41)	1 (1.75)		
	23 n.r.	15 n.r.	8 n.r.		
**Headache specialist: Have you had professional advice about your headache in the last year?**					
**No**	34 (70.83)	29 (74.36)	5 (55.56)	1.251	0.263
**Yes**	14 (29.17)	10 (25.64)	4 (44.44)		
	113 n.r.	57 n.r.	56 n.r.		
**Primary care doctor: Have you had professional advice about your headaches in the last year?**					
**No**	25 (52.08)	21 (53.85)	4 (44.44)	0.259	0.611
**Yes**	23 (47.92)	18 (46.15)	5 (55.56)		
	113 n.r.	57 n.r.	56 n.r.		
**Has a doctor ever given you a diagnosis for this headache?**					
**No**	122 (75.78)	70 (72.92)	52 (80.00)	1.059	0.303
**Yes**	39 (24.22)	26 (27.08)	13 (20.00)		
	0 n.r.	0 n.r.	0 n.r.		
**Because of your headaches, have you had a MRI scan in the last year?**					
**Yes**	12 (31.58)	9 (34.62)	3 (25.00)	0.351	0.553
**No**	26 (68.42)	17 (65.38)	9 (75.00)		
	123 n.r.	70 n.r.	53 n.r.		
**Because of your headaches, have you had a CT scan in the last year?**					
**Yes**	7 (35.00)	4 (15.38)	3	0.505	0.447
**No**	31 (65.00)	22 (84.62)	9		

	123 n.r.	70 n.r.	53 n.r.		
**Because of your headaches, have you had X rays of the neck in the last year?**<br>**Yes**<br>**No**	6 (15.79)<br>32 (84.21)<br>123 n.r.	4 (15.38)<br>22 (84.62)<br>70 n.r.	2 (16.67)<br>10 (83.33)<br>53 n.r.	0.10	0.920
**How bad is this headache usually?**<br>**Not Bad**<br>**Bad**<br>**Very Bad**	68 (42.24)<br>80 (49.69)<br>13 (8.07)<br>0 n.r.	36 (37.50)<br>51 (53.13)<br>9 (9.38)<br>0 n.r.	32 (49.23)<br>29 (44.62)<br>4 (6.15)<br>0 n.r.	2.326	0.313
	**all mean (SD)**	**females mean (SD)**	**males mean (SD)**	**Mann-Whitney-U**	**P value**
**HADS Depression**	5.75 (± 0.49)<br>38 n.r.	5.99 (± 5.55)<br>24 n.r.	5.41 (± 4.98)<br>14 n.r.	1887.500	0.791
**HADS Anxiety**	7.11 (± 4.89)<br>34 n.r.	7.71 (± 4.81)<br>23 n.r.	7.30 (± 4.93)<br>11 n.r.	2330.500	0.072
